# Correction: Nistor et al. Primary Cardiac Intimal Sarcoma: Multi-Layered Strategy and Core Role of *MDM2* Amplification/Co-Amplification and *MDM2* Immunostaining. *Diagnostics* 2024, *14*, 919

**DOI:** 10.3390/diagnostics14121235

**Published:** 2024-06-12

**Authors:** Claudiu Nistor, Camelia Stanciu Gavan, Adelina Birceanu, Cezar Betianu, Mara Carsote, Anca-Pati Cucu, Mihaela Stanciu, Florina Ligia Popa, Adrian Ciuche, Mihai-Lucian Ciobica

**Affiliations:** 1 Department 4-Cardio-Thoracic Pathology, Thoracic Surgery II Discipline, “Carol Davila” University of Medicine and Pharmacy, 050474 Bucharest, Romania; claudiu.nistor@umfcd.ro (C.N.); adrian.ciuche@umfcd.ro (A.C.); 2 Thoracic Surgery Department, “Dr. Carol Davila” Central Military Emergency University Hospital, 010242 Bucharest, Romania; camigavan@gmail.com (C.S.G.); anca-pati.cucu@drd.umfcd.ro (A.-P.C.); 3 Pathology and Immunohistochemistry Laboratory, Pathoteam Diagnostic, 051923 Bucharest, Romania; adelinabirceanu@gmail.com; 4 Department of Interventional Imaging, “Doctor Carol Davila” Central Military Emergency University Hospital, 010825 Bucharest, Romania; dr.cezarbetianu@gmail.com; 5 Department of Endocrinology, “Carol Davila” University of Medicine and Pharmacy, 050474 Bucharest, Romania; 6 Department of Clinical Endocrinology V, C.I. Parhon National Institute of Endocrinology, 020021 Bucharest, Romania; 7 PhD Doctoral School, “Carol Davila” University of Medicine and Pharmacy, 020021 Bucharest, Romania; 8 Department of Endocrinology, Faculty of Medicine, “Lucian Blaga” University of Sibiu, 550024 Sibiu, Romania; mihaela.stanciu@yahoo.com; 9 Department of Physical Medicine and Rehabilitation, Faculty of Medicine, “Lucian Blaga” University of Sibiu, 550024 Sibiu, Romania; florina.popa@yahoo.com; 10 Department of Internal Medicine and Gastroenterology, “Carol Davila” University of Medicine and Pharmacy, 020021 Bucharest, Romania; lucian.ciobica@umfcd.ro; 11 Department of Internal Medicine I and Rheumatology, “Dr. Carol Davila” Central Military University Emergency Hospital, 010825 Bucharest, Romania

Camelia Stanciu Gavan, Adelina Birceanu, and Cezar Betianu were not included as authors in the original publication [1]. The new addresses for Adelina Birceanu and Cezar Betianu are Affiliations 3 and 4, respectively. Additionally, all three authors have provided their email addresses. The corrected Author Contributions statement appears here. The authors state that the scientific conclusions are unaffected. This correction was approved by the Academic Editor. The original publication was also updated.
